# Bedside Ocular Ultrasound Diagnosis of a Traumatic Lens Dislocation

**DOI:** 10.7759/cureus.14666

**Published:** 2021-04-24

**Authors:** Andrew Glickman, Brian Szczucki, Eric J Kalivoda, Anthony Furiato, Gabriel Cabrera

**Affiliations:** 1 Emergency Medicine, HCA Healthcare/University of South Florida Morsani College of Medicine GME Consortium: Brandon Regional Hospital, Brandon, USA

**Keywords:** point-of-care ultrasound, ultrasonography, emergency ultrasound, eye trauma, lens dislocation, ectopia lentis, emergency department

## Abstract

Rapid identification of ophthalmologic emergencies can be challenging in the ED, and a missed or delayed diagnosis may have vision-threatening consequences. The application of ocular point-of-care ultrasound (POCUS) by the emergency physician (EP) can facilitate the timely recognition of a myriad of emergent eye conditions. This report describes a case in which EP-performed POCUS established the prompt diagnosis of a traumatic lens dislocation in a patient with chronic vision changes.

## Introduction

Ophthalmologic complaints represent about 1.5% of all ED visits, with only about half of these ED encounters deemed emergent [1,2]. Approximately 34% of eye-related ED visits are associated with trauma [2]. The diagnosis of traumatic ophthalmic emergencies can typically be challenging in the ED due to nonspecific visual symptoms with a difficult or limited physical examination [3]. Prompt consultation with an ophthalmologist and/or the availability of advanced imaging such as CT can oftentimes be delayed or not readily available. Nevertheless, it is critical that the emergency physician (EP) rapidly identify potentially vision-threatening conditions. Ocular point-of-care ultrasound (POCUS) offers an accurate bedside modality for the EP to diagnose several eye emergencies [4-7]. Previous reports have highlighted the utility of ocular POCUS to recognize lens dislocation after acute blunt eye trauma [8-12]. This case report describes the diagnosis of a traumatic lens dislocation with EP-performed ocular POCUS in a patient with blurry vision over several months.

## Case presentation

**Figure 1:**
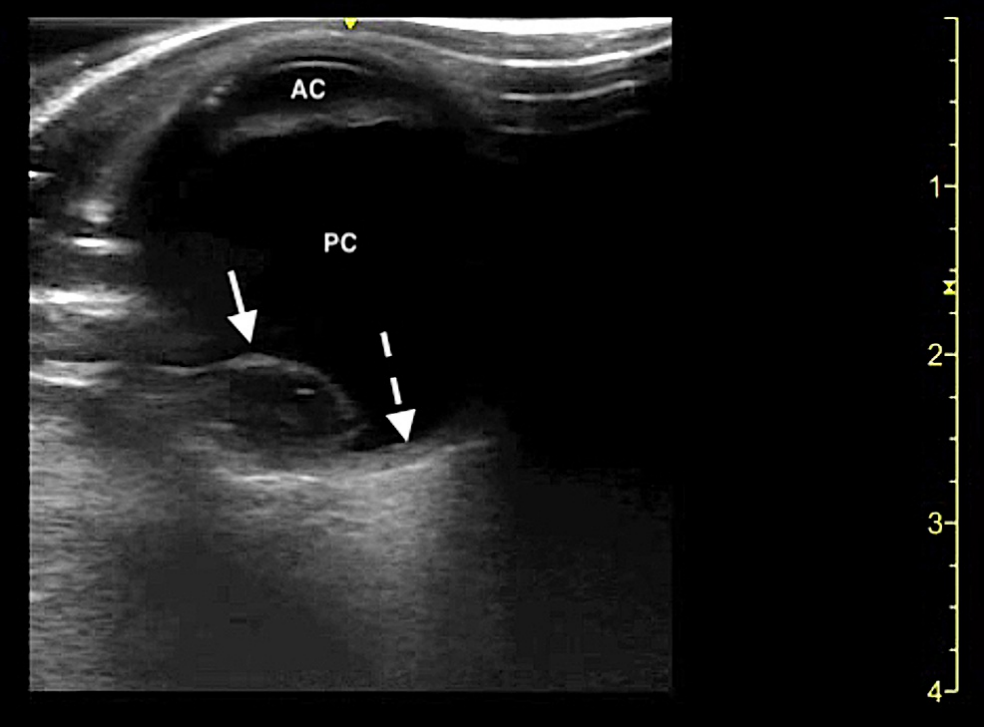
Ocular point-of-care ultrasound demonstrating a posteriorly dislocated lens. AC: Anterior chamber; PC: Posterior chamber; Dislocated lens (solid arrow); Retina (dotted arrow).

A 54-year-old non-domiciled male with a past medical history significant for hypertension and polysubstance abuse presented to the ED with a chief complaint of blurry vision in his right eye. The patient stated that he has been experiencing these symptoms after he was assaulted about six months prior with head and facial trauma. He denied eye pain, double vision, flashers, floaters, or eye discharge. He denied any foreign body sensation or the potential for an intraocular foreign body. The patient also denied any history of myopia or hyperopia requiring corrective vision assistance. Initial vital signs upon ED presentation were unremarkable. The patient was alert and oriented, no periorbital ecchymoses or head/facial trauma was present, and no focal neurological deficits were appreciated. The patient repeatedly declined a complete eye examination; therefore, the treating EPs were unable to obtain a visual acuity, fluorescein staining of the cornea, or intraocular pressures. A limited ocular exam was performed which demonstrated pupils that were equal, round, and reactive to light and extraocular movements were intact. There was no conjunctival injection, hyphema, hypopyon, or signs of gross globe injury. He was able to visualize motion only with the right eye and had normal vision with the left eye. The differential diagnosis of vision changes in the setting of traumatic eye injury included but was not limited to retinal detachment, vitreous hemorrhage/detachment, retrobulbar hematoma, and lens subluxation/dislocation. Ocular POCUS using a high-frequency linear transducer was the initial diagnostic modality chosen by the emergency medicine resident physicians. The patient’s right eye was covered with a sterile transparent medical dressing prior to beginning the study, and a scanning technique was performed as previously described [6,7]. POCUS revealed a hyperechoic, oval-shaped structure displaced into the posterior chamber of the right eye, consistent with a posterior lens dislocation (Figure 1).

**Figure 2:**
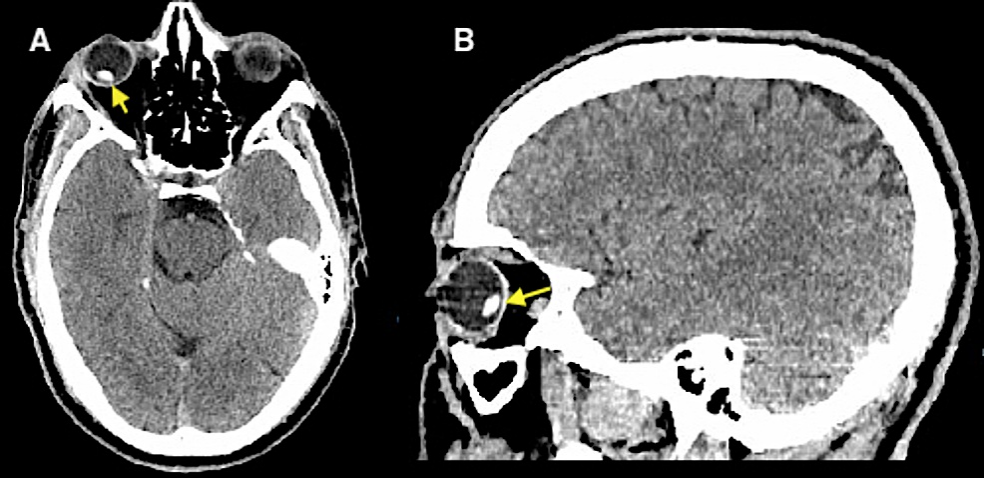
CT demonstrating a posterior lens dislocation of right orbit. A: Axial plane; B: Sagittal plane; Dislocated lens (yellow arrows).

CT of the brain was subsequently obtained and confirmed the findings suggested on ocular POCUS of a focal hyperdense 9 x 5 mm structure within the posterior aspect of the right globe likely representative of a displaced lens (Figure 2).

Given the chronic nature of the patient’s visual complaint, ophthalmology was not consulted emergently in the ED. The patient was counseled regarding the diagnosis of lens dislocation and the need to follow up promptly with an ophthalmologist. At the time of discharge, a referral to an outpatient ophthalmology clinic was provided.

## Discussion

Lens dislocations are a relatively uncommon etiology of acute traumatic vision changes predominantly caused by blunt eye trauma, which may develop significant complications such as worsening vision loss, cataract formation, retinal detachment, or glaucoma [3,5,13]. In the setting of eye trauma, the zonular fibers that hold the lens in place become disrupted, leading to the displacement of the lens into the anterior or posterior chamber [3,9-10]. Patients may present with pain in the affected eye, blurry vision, or vision loss [3]. The treatment of lens dislocation depends on the direction of lens displacement [3,12]. Anterior lens dislocations require emergent surgical correction if causing acute angle-closure glaucoma, whereas posterior lens dislocations can be managed by ophthalmology on an urgent basis [3,10,12]. Failure of conservative management or development of complications such as uveitis or glaucoma indicates the need for surgical correction of a posterior lens dislocation [3,12].

Ocular POCUS has emerged as an indispensable tool for EPs in the bedside diagnosis of a multitude of eye emergencies, including intraocular foreign bodies, retinal hemorrhage/detachments, vitreous hemorrhage/detachments, retrobulbar hematomas, papilledema, optic nerve evaluation of increased intracranial pressure, and lens subluxations/dislocations [4-7,13-15]. There are limited studies on the diagnostic accuracy of POCUS for the identification of lens dislocations, however, sensitivity and specificity of 97% and 99%, respectively, have been reported [14,15]. While CT imaging is the gold-standard diagnostic modality in the evaluation of globe trauma, it may not be readily available in all acute care settings [16]. POCUS is an important first-line, non-invasive adjunct to the clinical ophthalmic exam [16]. It enables the prompt recognition of traumatic pathology to the eye, including lens dislocation. This case illustrates the dynamic role of EP-performed ocular POCUS for the early ED diagnosis of traumatic lens dislocation.

## Conclusions

Timely recognition of eye emergencies can often be difficult in the ED setting. Implementing the findings of EP-performed ocular POCUS can expedite ophthalmology consultation when clinically indicated. Future studies are warranted to investigate the clinical impact of EP-performed ocular POCUS for traumatic lens dislocations and other emergent ophthalmologic conditions.
